# Hydrogen Sulfide Improves Drought Tolerance in *Arabidopsis thaliana* by MicroRNA Expressions

**DOI:** 10.1371/journal.pone.0077047

**Published:** 2013-10-23

**Authors:** Jiejie Shen, Tongji Xing, Huihong Yuan, Zhiqiang Liu, Zhuping Jin, Liping Zhang, Yanxi Pei

**Affiliations:** 1 School of Life Science, Shanxi University, Taiyuan, Shanxi, PR China; 2 College of Life Science, Zhejiang University, Hangzhou, Zhejiang, PR China; 3 Department of Biology, Appalachian State University, Boone, North Carolina, United States of America; 4 School of Chemical Engineering and Environment, North University of China, Taiyuan, Shanxi, PR China; CNR, Italy

## Abstract

Hydrogen sulfide (H_2_S) is a gasotransmitter and plays an important role in many physiological processes in mammals. Studies of its functions in plants are attracting ever growing interest, for example, its ability to enhance drought resistance in *Arabidopsis*. A general role of microRNAs (miRNAs) in plant adaptive responses to drought stress has thereby increased our interest to delve into the possible interplay between H_2_S and miRNAs. Our results showed that treating wild type (WT) *Arabidopsis* seedlings with polyethylene glycol 8000 (PEG8000) to simulate drought stress caused an increase in production rate of endogenous H_2_S; and a significant transcriptional reformation of relevant miRNAs, which were also triggered by exogenous H_2_S in WT. When *lcd* mutants (with lower H_2_S production rate than WT) were treated with PEG8000, they showed lower levels of miRNA expression changes than WT. In addition, we detected significant changes in target gene expression of those miRNAs and the corresponding phenotypes in *lcd*, including less roots, retardation of leaf growth and development and greater superoxide dismutase (SOD) activity under drought stress. We thereby conclude that H_2_S can improve drought resistance through regulating drought associated miRNAs in *Arabidopsis*.

## Introduction

Hydrogen sulfide (H_2_S) is emerging as an important endogenous gasotransmitter along with nitric oxide (NO) and carbon monoxide (CO) in eukaryotic organisms [1]–[3]. It has been implicated in regulating vasodilatation, smooth muscle relaxation, and cardio-protective processes in mammals [4]. In plants such studies are still at their beginning stages though they are attracting ever-growing attention. Currently H_2_S has been reported to participate in various physiological processes to improve drought resistance [5], [6]; increase longevity of cut flowers [7]; alleviate boron toxicity in cucumber seedlings [8]; alleviate cadmium induced oxidative damage in alfalfa seedling roots [9] and in *Escherichia coli* [10]; induce heat tolerance in tobacco suspension cultured cells [11]; enhance salt tolerance in alfalfa seed germination [12], etc.

To date, coding genes for several H_2_S-generating enzymes have been reported in different plant species. *l-cysteine* (*Cys*) *desulfhydrase* (*LCD*, At3g62130) and *d-Cys desulfhydrase 1* (*DCD1*, At1g48420) code for two classes of such enzymes that decompose l- and d-Cys into H_2_S, ammonia (NH_3_) and pyruvate [13]. *d-Cys desulfhydrase 2* (*DCD2*, At3g26115) is responsible for catalyzation of the decomposition of l- and d-Cys into H_2_S [14]. Another Cys desulfuration reaction catalyzed by the l-Cys desulfurases occurs in iron-sulfur cluster biosynthesis and involves the formation of l-Ala and elemental sulfur or H_2_S from Cys. Their coding genes are known as *NFS1* (At5g65720) and *NFS2* (At1g08490). Álvarez *et al.* reported that *DES1* (At5g28030) also codes for enzymes that catalyze the formation of H_2_S with l-Cys as substrate [15].

MicroRNAs (miRNAs) are a class of single-stranded non-coding RNAs that range in length from roughly 18–25 nucleotides, and are encoded by endogenous miRNA genes [16]. They have been reported to be involved in plant development, signal transduction, protein degradation and their own biogenesis regulation. In particular, miRNAs are known to regulate plant responses to a variety of biotic and abiotic stresses including drought, cold, salinity and bacterial infection [17]. In *Arabidopsis*, *miR156*, *miR158*, *miR159*, *miR165*, *miR167*, *miR168*, *miR169*, *miR171*, *miR319*, *miR393*, *miR394* and *miR396* are drought-responsive. Under drought stress, *miR167*, *miR393* and *miR396* are upregulated, *miR169* is downregulated and *miR398* is differentially regulated [17]. Jay *et al.* reported that *miR167* targets *auxin response factor 8* (*ARF8*) [18], which is involved in determining hypocotyl length, stamen development, and light signal transduction pathways [19]. *miR393* targets *transport inhibitor response 1* (*TIR1*), *auxin signaling F-box proteins 1*, *2* and *3* (*AFB1*, *AFB2* and *AFB3*) [20], which are involved in determining the length of the main root and hypocotyl and the number of lateral roots [21]. *miR396* targets growth-regulating factor *coding genes GRF1*, *GRF2*, *GRF3*, *GRF4*, *GRF7*, *GRF8* and *GRF9*, which play an important role in leaf growth and development [22]. *miR398* targets superoxide dismutase (SOD) coding genes. SOD is a major reactive oxygen species (ROS) scavenging enzyme and is also known as CSD1 in the cytoplasm and CSD2 in the chloroplast [23].

In a previous study, we found that H_2_S interacts with ABA in the stomatal regulation of drought stress in *Arabidopsis* [5]. Jin *et al.* reported that H_2_S upregulates several drought responsive genes including *dehydration-responsive element-binding protein 2A* and *2B* (*DREB2A* and *DREB2B*), *responsive to desiccation 29A* (*RD29A*) and *C-repeat-binding factor 4* (*CBF4*) to improve drought resistance in *Arabidopsis* [6].

In this study, we treated both wild type (WT) and *lcd* (a mutant that has lower H_2_S production rate than WT) *Arabidopsis* seedlings with polyethylene glycol 8000 (PEG8000) as simulation of drought stress to study the effects of H_2_S on the expression of drought associated miRNAs and their target genes and on the changes of corresponding phenotypes.

## Materials and Methods

### Plant Growth and Treatments

Seeds of *Arabidopsis* ecotype Columbia (Col-0) were used in this study. Seeds of T-DNA insertion mutant of *lcd* (SALK_082099) were obtained from the Arabidopsis Biological Resource Center (ABRC, http://www.arabidopsis.org/abrc/) [5]. For each experiment, seeds were incubated for 4 days at 4°C, sterilized in 75% (v:v) ethanol solution for 30 sec and in 6% (v:v) sodium hypochlorite solution for 9 min, then placed in a growth chamber at 23±1°C on ½ MS (Murashige-Skoog) medium at about 160 µmol photons m^−2^ s^−1^ for 14 d with a 16/8 h (light/dark) photoperiod.

After 14 days, seedlings were carefully removed with their roots immersed in water or PEG8000 serial solution. WT seedlings were treated with the following four treatments: 50 µmol L^−1^ NaHS [6] for 0, 3, 6, 12 h; 0, 20, 50, 100 µmol L^−1^ NaHS for 12 h; 0.1 g ml^−1^ PEG8000 solution (based on the data in our lab previously) for 0, 1, 2, 4, 8 h; 0, 0.05, 0.1, 0.2, 0.4 g ml^−1^ PEG8000 solution for 2 h.

### Reverse Transcription (RT)-PCR Analysis

Total RNAs were extracted from 14-d old seedlings in ½ MS medium. RT reactions were performed in 20 µl system using 3 µg RNA by M-MLV (NEB). RT-PCR conditions for elongation factor 1-α gene (*EF1-α*) amplification were as follows: 94°C for 1 min, 94°C for 1 min, 66°C for 30 sec, 72°C for 50 sec, 35 cycles, and 72°C for 10 min [24]. For target gene amplification, essentially the same conditions were used except the number of PCR cycles and annealing temperatures were varied (see Table S1).

The cDNAs above were used as templates to determine expression levels of miRNAs and target genes with quantitative real-time PCR (qRT-PCR). The primers used for qRT-PCR are listed in Table S1. Analyses were performed using the BioRad Real-Time System (CFX96TM C1000 Thermal Cycler, Singapore). In the relative quantification analysis, *ACTIN* was used as a reference gene to normalize expression values. All experiments were repeated three times along with three independent repetitions of the biological experiments and the results were analyzed using the delta-delta threshold cycle method [25].

### Measurement of H_2_S Production Rate

H_2_S production rate was measured according to Jin *et al.* [6]. The extraction of total protein amount in 14-day-old plants was according to Pei *et al.* [24]. Protein content was determined according to Bradford [26].

### Observation of Phenotype and Determination of Relevant Physiological Indexes

Measurement of roots and leaf growth and development was as follows. WT and *lcd* seedlings were cultured for 26 days under the same conditions as above. Then the length and the number of roots were measured and statistically analyzed; the growth and development of leaves were also observed.

SOD activities and malondialdehyde (MDA) content were measured according to Jiang *et al.* [27]. H_2_O_2_ content was measured according to Alexieva *et al.* [28].

### Statistical Analysis

Analyses of variance were conducted to determine treatment differences using SPSS (version 17, IBM SPSS, Chicago, IL). We used the LSD multiple range tests to evaluate significant differences among the treatments (*P<0.05*).

## Results

### Effects of PEG8000 on Expression Levels of H_2_S-generating Enzymes and Production Rate of H_2_S in WT Seedlings

**Figure 1:**
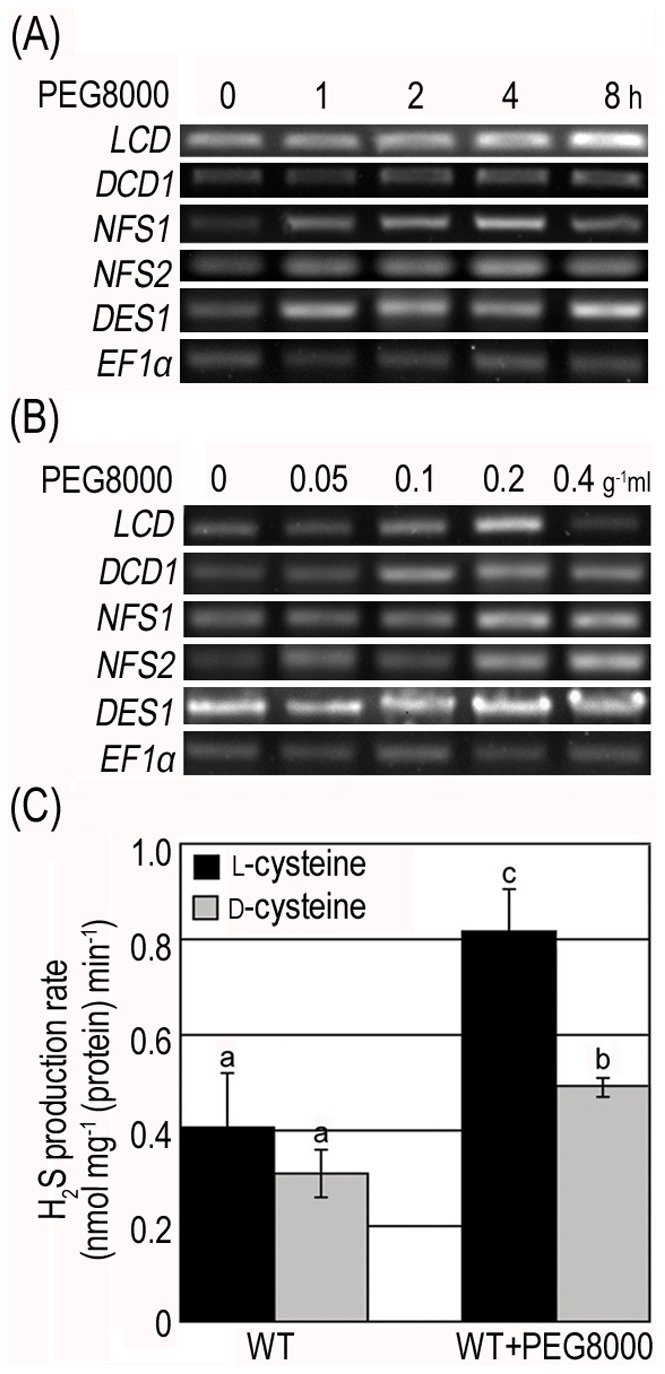
Effects of PEG8000 on the expression of genes controlling H_2_S generation and on H_2_S production rate in WT seedlings. (**A**) Expression detection of H_2_S generating critical enzymes coding genes in WT seedlings treated with 0.1 g ml^−1^ PEG8000 for 0, 1, 2, 4, 8 h; (**B**) Expression detection of H_2_S generating critical enzymes coding genes in WT seedlings treated 2 h with 0, 0.05, 0.1, 0.2, 0.4 g ml^−1^ PEG8000. *EF1-α* was used as an internal control of RT-PCR; (**C**) Endogenous H_2_S production rate of WT seedlings treated with 0.2 g ml^−1^ PEG8000 for 2 h. Results are shown as mean ± SE (n = 3 independent experiments). Letter numbers indicate significant differences between treatments and substracts (*P<0.05*).

Expression levels of H_2_S-generating enzymes (*LCD*, *DCD1*, *NFS1*, *NFS2* and *DES1*) were determined by RT-PCR. The accumulation of gene transcripts mentioned above increased as time progressed and as PEG8000 concentration was elevated (Figure 1A and 1B). *LCD* was an anomaly as its transcripts reached a peak when treated with 0.2 g ml^−1^ PEG8000 (Figure 1B). In addition, measurement of H_2_S product rate in WT treated with 0.2 g ml^−1^ PEG8000 showed that the decomposing rate of l- and d-Cys into H_2_S significantly increased within 2 h upon the initiation of treatment (Figure 1C). These results established a significant correlation between drought stress and the production of both H_2_S transcripts and H_2_S emission.

### Effect of PEG8000 on the Expression of Drought Associated miRNAs in WT Seedling

**Figure 2:**
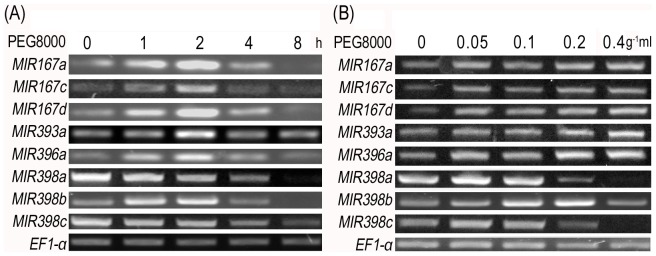
RT-PCR detection of miRNAs transcription in WT seedlings under PEG8000 stress. (**A**) miRNAs transcription detection in WT seedlings treated with 0, 1, 2, 4, 8 h using 0.01 g ml^−1^ PEG8000 treatment; (**B**) miRNAs transcription detection in WT seedlings after 2 h using different PEG8000 concentration treatments at 0, 0.05, 0.1, 0.2, 0.4 g ml^−1^ PEG8000. *EF1-α* was used as an internal control of RT-PCR.

Khraiwesh *et al.* reported that miRNAs play a role in plant responses to drought stress [17]. Therefore we treated WT *Arabidopsis* with PEG8000 to simulate drought stress in order to determine expression-level changes of drought associated miRNAs by RT-PCR. The results showed an accumulation of *MIR167a*, *MIR167c*, *MIR167d*, *MIR393a* and *MIR396a* transcripts as time progressed until they reached a maximum at 2 h into the treatment, after which they started decreasing (Figure 2A). Thus a PEG8000 treatment for 2 h was selected for later experiments. We then treated WT seedlings with different concentrations of PEG8000 (0, 0.05, 0.1, 0.2, 0.4 g ml^−1^) and found that higher expression of the miRNAs was induced by increased PEG8000 concentration. However the expression levels reached a plateau when treated with 0.2 g ml^−1^ solution (Figure 2B). The 0.2 g ml^−1^ PEG8000 treatment was therefore selected for ensuing experiments. *MIR398a* and *MIR398c* transcripts decreased as time progressed, While *MIR398b* transcripts increased as time progressed, until they reached a maximum at 2 h into the treatment, after which started decreasing (Figure 2A); transcripts first increased and then decreased as PEG8000 concentration increased (Figure 2B). These results collectively indicated that the expression levels of specific miRNAs corresponded to drought stress caused by PEG8000.

### Effect of H_2_S on the Drought Associated miRNAs Expression in WT Seedling

**Figure 3:**
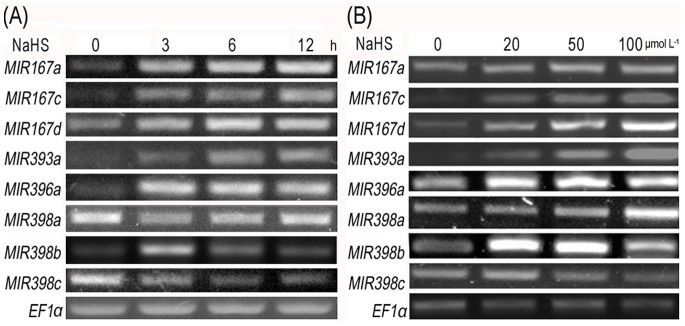
NaHS effects on miRNAs expression in WT seedlings. (**A**) miRNAs expression in WT seedlings treated with 50 µmol L^−1^ NaHS for 0, 3, 6, 12 h; (**B**) miRNAs expression in WT seedlings treated with 0, 20, 50, 100 µmol L^−1^ NaHS for 12 h. *EF1-α* was used as an internal control.

To further validate the above conclusions, we treated WT seedlings with 50 µmol L^−1^ NaHS for 0, 3, 6, 12 h. The results showed that exogenous H_2_S induced a common pattern of transcript accumulation of *MIR167a*, *MIR167c*, *MIR167d*, *MIR393a* and *MIR396a* as time progressed (Figure 3A). NaHS for 12 h was thereby selected in the following experiments. In comparison, *MIR398a* and *MIR398b* were first downregulated and then upregulated; *MIR398c* was downregulated during the 12 h period. When the seedlings were treated with 0, 20, 50 µmol L^−1^ NaHS for 12 h, all miRNAs above were upregulated in a dose-dependent manner except for *MIR398b* and *MIR398c* (Figure 3B). When treated with 100 µmol L^−1^ NaHS, expression of these miRNAs except for *MIR398b* and *MIR398c* showed no significant increase and therefore we chose 50 µmol L^−1^ for the following experiments. *MIR398b* and *MIR398c* set themselves apart by exhibiting an up-down-up regulatory pattern of their transcripts (Figure 3B). These results indicated that the expression of the related miRNAs was affected by exogenous H_2_S treatment.

### H_2_S Responds to Drought Stress by Regulating miRNAs in *Arabidopsis*

**Figure 4:**
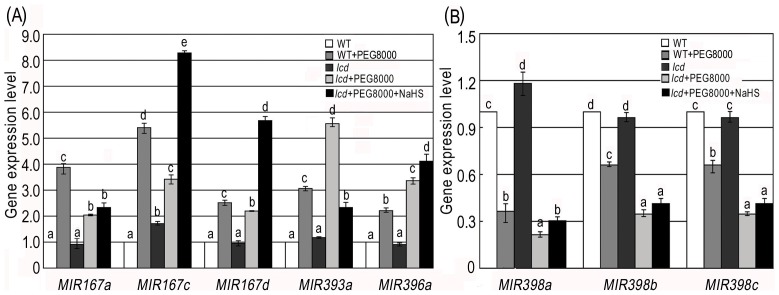
PEG8000 and NaHS effects on miRNAs in WT and *lcd* plants. (**A**) *MIR167a*, *MIR167c*, *MIR167d*, *MIR393a* and *MIR396a* expressions in WT and *lcd* treated with 50 µmol L^−1^ NaHS and 0.2 g ml^−1^ PEG8000. *lcd* was pre-treated with 50 µmol L^−1^ NaHS for 12 h and 0.2 g ml^−1^ PEG8000 for 2 h; (**B**) *MIR398a*, *MIR398b* and *MIR398c* expression in WT and *lcd* treated with 50 µmol L^−1^ NaHS and 0.2 g ml^−1^ PEG8000. The same treatments were applied as in (**A**). *ACTIN* was used as an internal control in qRT-PCR. Results are shown as mean ± SE (n = 3 independent experiments). Letter numbers indicate significant differences between treatments within one gene (*P<0.05*).

As suggested by results from the above experiments, we made the assumption that the H_2_S signal was intensified by drought stress in *Arabidopsis* WT seedlings and that H_2_S further regulated plant responses to drought through the miRNA pathway. To validate this, we introduced *lcd* mutants and treated them with 50 µmol L^−1^ NaHS and 0.2 g ml^−1^ PEG8000 separately. *lcd* mutants are lack of the critical H_2_S generating enzyme LCD and their H_2_S production rate is determined to be 40% of the WT. Results from qRT-PCR showed elevated expression levels of *MIR167a*, *MIR167c*, *MIR167d*, *MIR393a* and *MIR396a* (Figure 4A) and decreased expression levels of *MIR398a*, *MIR398b* and *MIR398c* (Figure 4B) in both *lcd* and WT under PEG8000 treatment compared with non-treated plants. When PEG8000 treated *lcd* mutants were compared with PEG8000 treated WT, *lcd* showed a lower expression level of *MIR167a*, *MIR167c*, *MIR167d*, *MIR398a*, *MIR398b* and *MIR398c* and a higher expression level of *MIR393a and MIR396a.* We conclude that miRNA expression in general is lower in PEG8000 treated *lcd* than PEG8000 treated WT. However when deficient endogenous H_2_S production was rescued by NaHS supply, we again observed an accumulation of relevant miRNA transcripts (Figure 4), which confirmed H_2_S regulates miRNAs to improve tolerance to drought stress in *Arabidopsis*.

### Expression Changes of Drought Associated miRNAs Target Genes under PEG8000 Stress and NaHS Treatment in *Arabidopsis*

**Figure 5:**
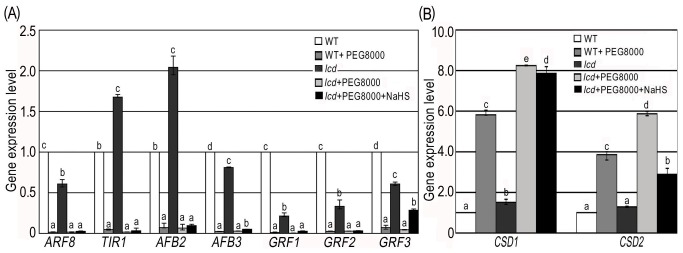
Target gene expressions in WT and *lcd* plants treated with PEG8000 and NaHS. (**A**) *ARF8*, *TIR1*, *AFB2*, *AFB3*, *GRF1*, *GRF2* and *GRF3* expression in WT and *lcd* treated with 50 µmol L^−1^ NaHS and 0.2 g ml^−1^ PEG8000; (**B**) *CSD1* and *CSD2* expression in WT and *lcd* treated with 50 µmol L^−1^ NaHS and 0.2 g ml^−1^ PEG8000. The same treatments were applied as in Figure 4. *ACTIN* was used as an internal control in qRT-PCR. Results are shown as mean ± SE (n = 3 independent experiments). Letter numbers indicate significant differences between treatments within one gene (*P<0.05*).

We selected *ARF8* (target gene of *miR167*); *TIR1*, *AFB2* and *AFB3* (target genes of *miR393*); *GRF1*, *GRF2* and *GRF3* (target genes of *miR396*); *CSD1* and *CSD2* (target genes of *miR398*) to determine possible transcriptional changes of drought-associated miRNA target genes. Results from qRT-PCR showed significant lower expression of *ARF8*, *TIR1*, *AFB2*, *AFB3*, *GRF1*, *GRF2* and *GRF3* (Figure 5A), and significant higher expression of *CSD1*and *CSD2* (Figure 5B) in *lcd* and WT, under PEG8000 treatment compared that without PEG8000. When *lcd* is compared to WT with PEG8000, *CSD1*and *CSD2* both had higher expression levels while other target genes showed no obvious difference. When *lcd* plants treated with NaHS and PEG8000 were compared to *lcd* plants treated with only PEG8000, *CSD1*and *CSD2* both had lower expression levels while other target genes (*AFB3*, *GRF3*, *CSD1* and *CSD2*) had higher abundance, which possibly offset the deficiency effects caused by lack of *LCD.* We may thereby conclude that H_2_S affects the expression of those downstream target genes by regulating miRNAs.

### Phenotype Observation Corresponding to the Target Genes of Drought Associated miRNAs

**Figure 6:**
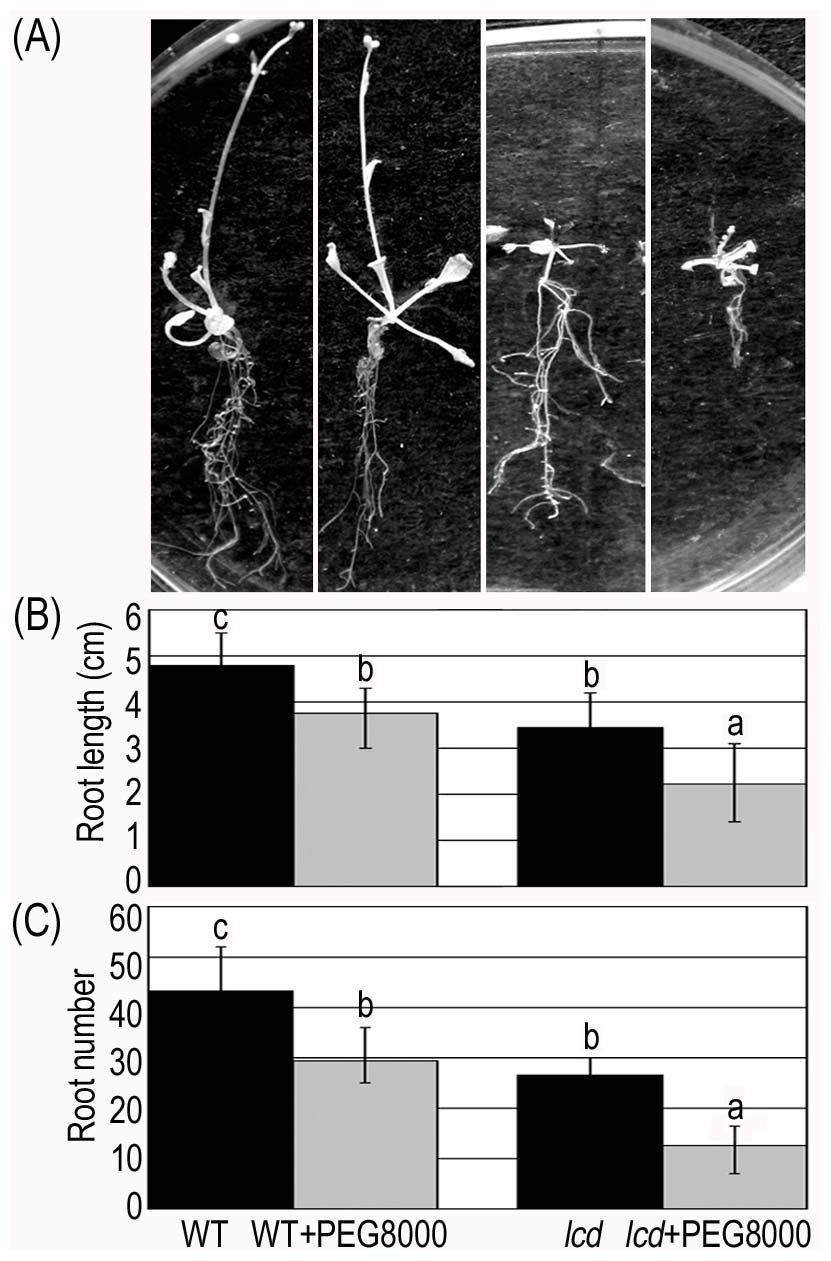
Root number and length in WT and *lcd* plants treated with PEG8000. (**A**) WT and *lcd* seedlings cultured in ½ MS and ½ MS containing PEG8000 for 26 day. (**B**) Length of roots. (**C**) Number of roots. Results are shown as mean ± SE (n = 3 independent experiments). Letter numbers indicate significant differences (*P<0.05*).

**Figure 7:**
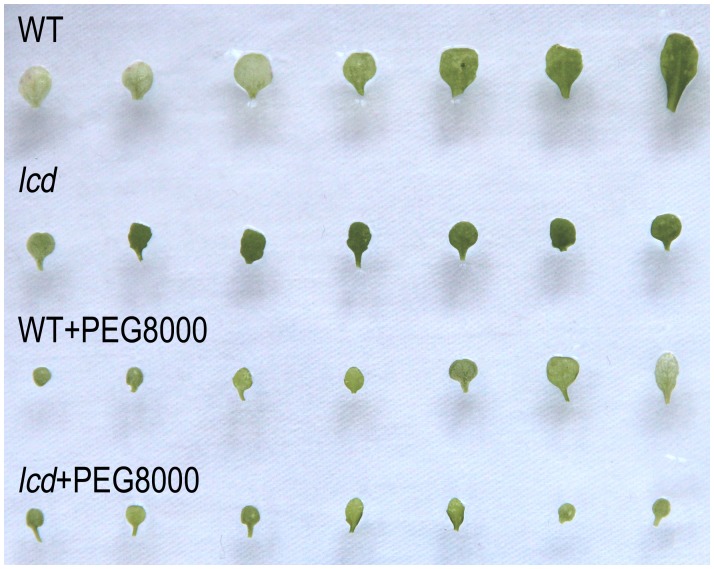
Leaf growth and development of WT and *lcd* plants treated with PEG8000. WT and *lcd* seedlings cultured in ½ MS and ½ MS containing PEG8000 for 26 day. Experiments are repeated three times.

**Figure 8:**
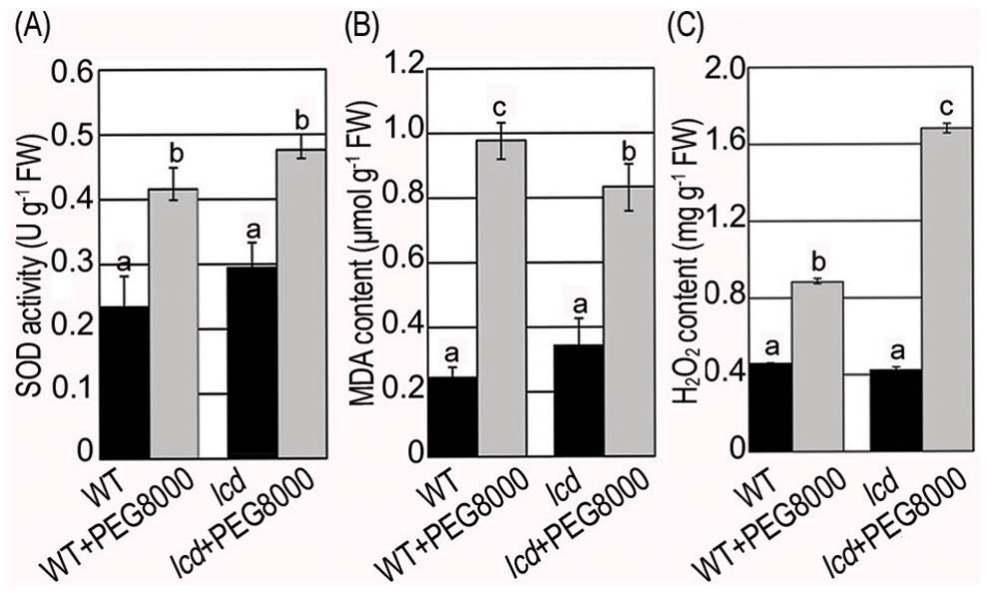
SOD activity, H_2_O_2_ content and MDA content in WT and *lcd* plants treated with PEG8000. (**A**) SOD activity was measured in WT and *lcd* plants treated with 0.2 g ml^−1^ PEG8000 for 2 h. One SOD unit was the amount of enzyme required to inhibit photoreduction of nitro blue tetrazolium chIoride by 50% at 25°C. SOD activity was expressed as U mg^−1^ (protein) min^−1^. (**B**) H_2_O_2_ content was measured in WT and *lcd* plants treated with 0.2 g ml^−1^ PEG8000 for 2 h. (**C**) MDA content was measured in WT and *lcd* plants treated with 0.2 g ml^−1^ PEG8000 for 2 h. Results are shown as mean ± SE (n = 3 independent experiments). Letter numbers indicate significant differences between treatmeats (*P<0.05*).

We observed the phenotypes corresponding to the drought associated miRNA target genes to further validate that H_2_S affects the expression of those downstream target genes by regulating miRNAs. According to previous research, *TIR1*, *AFB1*, *AFB2* and *AFB3* (targets of *miR393*) affect the growth of the main root and hypocotyl and the number of lateral roots [21]. The length and the number of roots decreased 28% and 32% in dehydrated WT, respectively (Figure 6B and 6C); in dehydrated *lcd* they decreased to a greater extent: 40% in the length and 52% in the number of roots (Figure 6). *GRF1*, *GRF2*, *GRF3*, *GRF4*, *GRF7*, *GRF8* and *GRF9* (targets of *miR396*) function primarily in leaf development and when overexpressed plants have lower densities of stomata [22]. The size of leaves decreased in both WT and *lcd* under PEG8000 but in *lcd* it decreased to a greater extent (Figure 7). *CSD1*and *CSD2* (targets of *miR398*) play an important role in scavenging activity of ROS (results shown in Figure 8) [23]; SOD enzyme activity increased in both WT and *lcd* under PEG8000 (Figure 8A); Similarly, H_2_O_2_ and MDA contents increased in both WT and *lcd* under PEG8000 and it is notable that MDA content increased to a greater extent in *lcd* compared with WT (Figure 8B and 8C).

In summary, *lcd* in comparison with WT under PEG8000 showed decreased root lengths, fewer roots, significantly smaller leaf sizes and increased antioxidant enzyme activities. These results confirmed our initial speculation that H_2_S affects the expression of those downstream target genes that respond to drought by regulating their corresponding miRNAs.

## Discussion

In our experiments a 50 µmol L^−1^ concentration of NaHS was selected based on the known physiological concentration range of H_2_S of 1 to 100 µmol detected in animals and plants [6]. To confirm 50 µmol L^−1^ as the proper H_2_S physiological concentration in *Arabidopsis*, we measured the MDA content in WT and *lcd* treated with NaHS and our results showed that the MDA contents in WT and *lcd* were not significantly different from corresponding controls (Figure S1). Therefore, 50 µmol L^−1^ of NaHS was an appropriate choice for this research.

**Figure 9:**
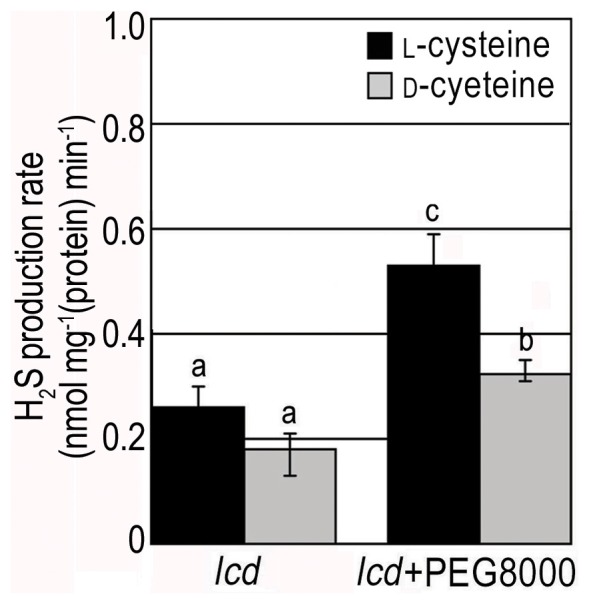
H_2_S generation rate in *lcd* plants treated with PEG8000. Endogenous H_2_S generation rate in *lcd* seedlings treated with 0.2 g ml^−1^ PEG8000 for 2 h. Results are shown as mean ± SE (n = 3 independent experiments). Letter numbers indicate significant differences between treatmeats and substracts (*P<0.05*).

After we found that H_2_S product rate in WT plant under drought stress was accelerated, we also measured the same index in *lcd* and detected a higher generation rate under drought stress than that without PEG8000 as well (Figure 9). We surmise that this is due to a complementary effect of other H_2_S generating enzymes such as DCD1, DCD2, DES1, NFS1 and NFS2. It is therefore highly probable that drought stress induces the expression of other H_2_S generating enzymes as well as LCD.

According to Goetz *et al.* [19], *ARF8* (target of *miR167*) prompts the elongation of hypocotyl and stamens during development, and regulates light signal transduction pathways. However we found that the length of the hypocotyl decreased in both WT and *lcd* under PEG8000 (date not shown), which complies with the observable decreased hypocotyl length of alfalfa [29] and *Pinus sylvestris* var. *mongolica* seeds [30] under drought conditions. This is possible since there are a number of factors regulating the growth of the hypocotyl when plants lack water. While *miR167* is responsible for hypocotyl growth, other factors such as the activation of α- and β-amylases [29], [31], and *Arabidopsis* AP2/DREB-type transcription factor [32] may suppress growth and cause an overall effect of decreased hypocotyl length.

In this paper we have shown that the expression of *MIR398a* and *MIR398c/MIR398b* first increased as PEG8000 concentration went up from 0 to 0.05 g ml^−1^/0.2 g ml^−1^ and then decreased (Figure 2B). This is consistent with the results from Trindade *et al.* [33] and Frazier *et* *al.* [34], however deviating from a commonly discovered negative correlation between miRNA transcripts and PEG8000 concentration [35], [36]. Therefore different miRNA species may have different sensitivity to PEG8000 concentrations. *miR398* target genes that code for the free radical scavenger SOD and these genes have been shown to be down-regulated during times of oxidative stress [36]. Therefore decreased *miR398* expression of plants exposed to higher PEG8000 concentrations might suggest that severe drought induced stress was created by an oxidative environment inside the *Arabidopsis* cells. However the exact mechanism of action remains unclear.”

Expression of *MIR167a*, *MIR167c*, *MIR167d*, *MIR398a*, *MIR398b and MIR398c* transcripts in *lcd* are significantly lower than WT under PEG8000 treatment while that of *MIR393a* and *MIR396a* are higher (Figure 4), which did not match the expression pattern of the miRNA target genes. However, eukaryotic organisms are complex and a certain signal transduction pathway for some intermediates can at the same time be involved in other physiological and biochemical reactions or other metabolic pathways in the cell. The overall effect could lead to unexpected results such as in our example, where the expression of some target genes did not match that of their miRNAs.

In order to detect any possible changes of antioxidant enzymes under drought conditions, we measured activity of two commonly known antioxidant enzymes, peroxidase (POD) and catalase (CAT), and found that both activities were significantly raised in both WT and *lcd* after treatment, which parallels the SOD activity we obtained (Figure S2). Using H_2_S against drought-induced oxidative stress might be a common process in various plant species [37].

**Figure 10:**
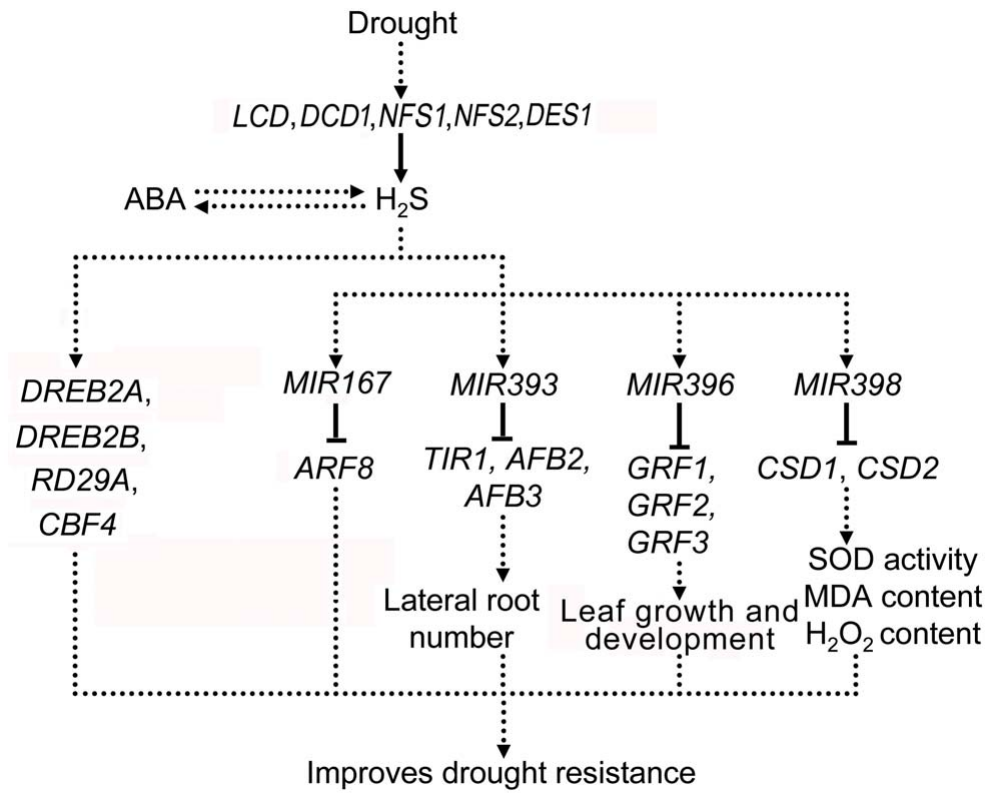
Summary of H_2_S regulating miRNAs in response to drought stress. Solid lines: direct effects; dotted lines: intermediates remain elusive; arrows: enhanced expression; hyphen: suppressed expression.

We propose a model based on the results described in this study and previous research [5], [6] in our lab, demonstrating the H_2_S regulating pathway in response to drought stress (Figure 10). Drought upregulates the expression levels of *LCD*, *DCD1*, *NFS1*, *NFS2* and *DES1* in order to produce more H_2_S which then interacts with ABA. On one hand, H_2_S directly regulates the expression of a series of drought responsive genes including *DREB2A*, *DREB2B*, *RD29A* and *CBF4*. On the other hand, H_2_S is involved in regulating the expression of drought associated miRNAs such as *miR167*, *miR393*, *miR396* and *miR398* and can therefore affect their target gene expressions and so to improve the tolerance of *Arabidopsis* to drought.

## Supporting Information

Figure S1 **The effect of NaHS on the content of MDA in WT and** ***lcd*** **plants.** The MDA content of WT and *lcd* seedlings were determined after being treated with 50 µmol L^−1^ NaHS for 12 h. Results shown are mean ± SE (n = 3 independent experiments). Letter numbers indicate significant differences between treatments (*P<0.05*).
(TIF)

Figure S2 **CAT activity and POD activity in WT and** ***lcd*** **plants treated with PEG8000.** (**A**) CAT activity was measured in WT and *lcd* plants treated with 0.2 g ml^−1^ PEG8000 for 2 h. One CAT unit was the amount of enzyme required to decompose 1 µmol of H_2_O_2_ min^−1^ at 25°C (pH 7.0). Consumption of H_2_O_2_ was measured as the decrease in absorbance at 240 nm. (**B**) POD activity was measured in WT and *lcd* plants treated with 0.2 g ml^−1^ PEG8000 for 2 h. One POD unit was the amount of enzyme required to decompose 1 µmol of H_2_O_2_ min^−1^ at 25°C (pH 7.0). Consumption of H_2_O_2_ was measured as the decrease in absorbance at 470 nm. Results shown are mean ± SE (n = 3 independent experiments). Letter numbers indicate significant differences between treatmeats (*P<0.05*).
(TIF)

Table S1 **List of all genes in the manuscript.**
(DOC)
