# Supplementary figures and images for: Hydrogen Sulfide Improves Drought Tolerance in Arabidopsis thaliana by MicroRNA Expressions
Source: PLoS One. 2013 Oct 23;8(10):e77047. doi: 10.1371/journal.pone.0077047 (PMC3806758; doi:10.1371/journal.pone.0077047)

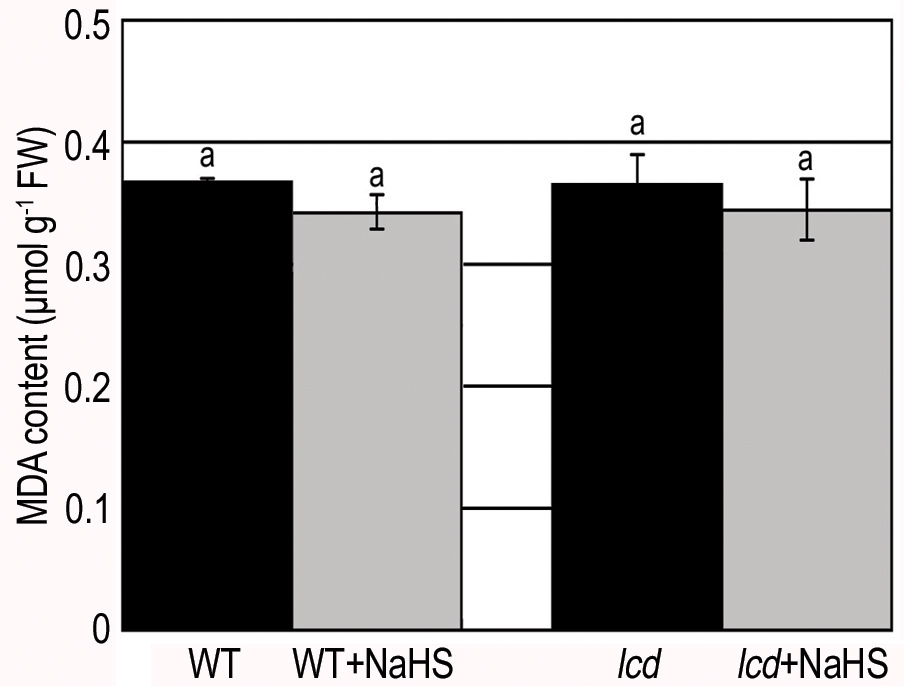

Supplement: Figure S1 — The effect of NaHS on the content of MDA in WT and lcd plants. The MDA content of WT and lcd seedlings were determined after being treated with 50 µmol L−1 NaHS for 12 h. Results shown are mean ± SE (n = 3 independent experiments). Letter numbers indicate significant differences between treatments (P<0.05). (TIF) [file pone.0077047.s001.tif]

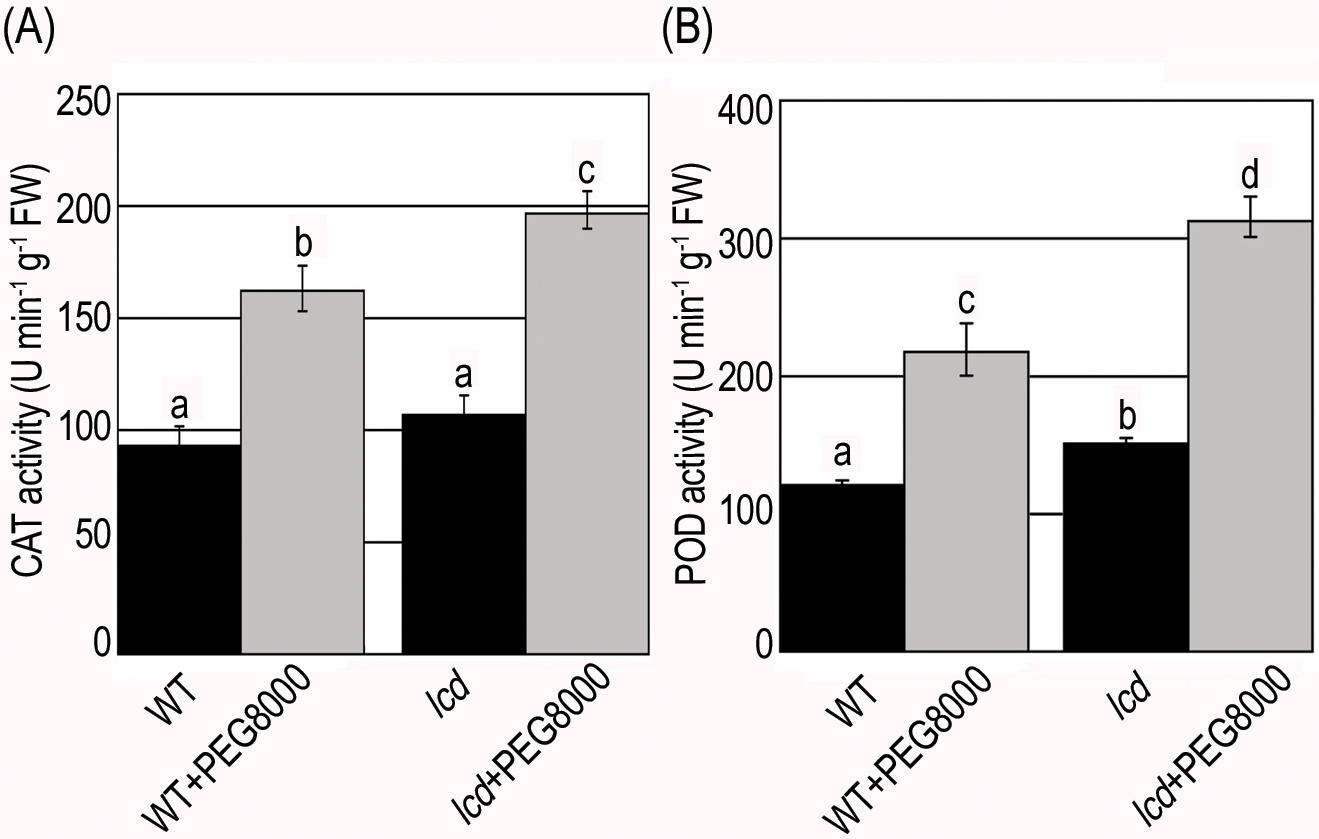

Supplement: Figure S2 — CAT activity and POD activity in WT and lcd plants treated with PEG8000. (A) CAT activity was measured in WT and lcd plants treated with 0.2 g ml−1 PEG8000 for 2 h. One CAT unit was the amount of enzyme required to decompose 1 µmol of H2O2 min−1 at 25°C (pH 7.0). Consumption of H2O2 was measured as the decrease in absorbance at 240 nm. (B) POD activity was measured in WT and lcd plants treated with 0.2 g ml−1 PEG8000 for 2 h. One POD unit was the amount of enzyme required to decompose 1 µmol of H2O2 min−1 at 25°C (pH 7.0). Consumption of H2O2 was measured as the decrease in absorbance at 470 nm. Results shown are mean ± SE (n = 3 independent experiments). Letter numbers indicate significant differences between treatmeats (P<0.05). (TIF) [file pone.0077047.s002.tif]
