# Supplementary material for: Hydrogen Sulfide Improves Drought Tolerance in Arabidopsis thaliana by MicroRNA Expressions
Source: PLoS One. 2013 Oct 23;8(10):e77047. doi: 10.1371/journal.pone.0077047 (PMC3806758; doi:10.1371/journal.pone.0077047)
Supplement: Table S1 — List of all genes in the manuscript. (DOC) [file pone.0077047.s003.doc]

**Table S1**. **List of all genes in the manuscript**

| Gene | Accession number | Primer pairs | Tm (^o^C) | PCR product (bp) | PCR cycles |
| --- | --- | --- | --- | --- | --- |
| *EF1-α* | At5g60390 | ATGCCCCAGGACATCGTGATTTCAT | 66 | 709 | 35 |
|  |  | TTGGCGGCACCCTTAGCTGGATCA |  |  |  |
| *ACTIN* | AT5G09810 | CTCAGCACCTTCCAACAGATGTGGA | 55 | 364 | 35 |
|  |  | CCAAAAAAATGAACCAAGGACCAAA |  |  |  |
| *MIR167a* | At3g22886 | GTGTAGTCAACTGTGTGCGTT | 53 | 234 | 35 |
|  |  | GCACAACTTGTTGCTCAGGT |  |  |  |
| *MIR167c* | At3g04765 | TTCATGCTACAATCATTAGCAGGT | 53 | 205 | 35 |
|  |  | AGTCGTCTTCATGTCTGTATGT |  |  |  |
| *MIR167d* | At1g31173 | GAGTTGTGGCCATTAAGAGCT | 53 | 216 | 35 |
|  |  | CTTCTTGTTAATGTTTGCTCTCTCCT |  |  |  |
| *MIR393a* | At2g39885 | CAAAGAGATAGCATGATCCAA | 53 | 214 | 35 |
|  |  | AAGAGGAACACGA TCCATTGAC |  |  |  |
| *MIR396a* | At2g10606 | AGGGTTTCGTCTGCTCTACAT | 53 | 242 | 35 |
|  |  | TCTGATTATG GAATCAATCACGCT |  |  |  |
| *MIR398a* | At2g03445 | AGAGAAGAACAACAGGAGGTGAAAT | 58 | 162 | 40 |
|  |  | GGTGAAAAAATGGAACAGGGGAGAT |  |  |  |
| *MIR398b* | At5g14545 | TAACAAGAAGATATCAATATATCA | 60 | 180 | 38 |
|  |  | ACCATTTGGTAAATGAGTAAAAGCCAGCC |  |  |  |
| *MIR398c* | At5g14565 | TCGAAACTCAAACTGTAACAGTCC | 57 | 240 | 40 |
|  |  | ATTTGGTAAATGAATAGAAGCCACG |  |  |  |
| *LCD* | At3g62130 | CGGGATCCATGGAGGCGGGAGAGCGGCGC | 55 | 1365 | 38 |
|  |  | ACGCGTCGACCTACAATGCAGGAAGGTTTTGAC |  |  |  |
| *DCD* | At1g48420 | CGGGATCCATGAGAGGACGAAGCTTGACAC | 55 | 1206 | 40 |
|  |  | ACGCGTCGACCTAGAACATTTTCCCAACACCAT |  |  |  |
| *NFS1* | At5g65720 | CATGCCATGGCGTCTAAGGTAATCTCTG | 59 | 1300 | 38 |
|  |  | ATCGGATCCGTGTTGAGACCATTGAATGTTC |  |  |  |
| *NFS2* | At1g08490 | CATGCCATGGAAGGTGTGGCTATGAAACTC | 57.3 | 1300 | 40 |
|  |  | CGGGATCCTTTGAAAGAGTTGAAGAAGCTCACAG |  |  |  |
| *DES1* | At5g28030 | CATGCCATGGAAGACCGCGTC | 60 | 972 | 38 |
|  |  | CCGGAATTCTCATTCAACTGGC |  |  |  |
| *CSD1* | At1g08830 | AGACGAAGCAAAAACATTCAGAGA | 55 | 204 | 40 |
|  |  | GGCCAGAAACTGTTCCACTC |  |  |  |
| *CSD2* | At2g28190 | CGTCTTCTCATTCCTCCTTCC | 55 | 417 | 40 |
|  |  | GGCATTGGCATTTATGTTTCC |  |  |  |
| *ARF8* | At5g37020 | AGATGTTTGCTATCGAAGGGTTGTTG | 55 | 106 | 40 |
|  |  | CCATGGGTCATCACCAAGGAGAAG |  |  |  |
| *TIR1* | At3g62980 | GCCTCTCTCTATCTGGCCTCTTGAC | 55 | 349 | 40 |
|  |  | AGGGCAGCTCTCTGGTCTCGAGTCC |  |  |  |
| *AFB2* | At3g26810 | AGATGCTCTCCATAGCCTTTGCAGG | 55 | 349 | 40 |
|  |  | TGGCGGCGCATCCATTCTTGTCCCA |  |  |  |
| *AFB3* | At1g12820 | AGCTCGAGATGCTTTCGATAGCTTTTG | 55 | 279 | 40 |
|  |  | TCATTCTGTTCCATCCCATTATTCTCA |  |  |  |
| *GRF1* | At2g22840 | GATTACCTCCCATGGGAAAA | 55 | 423 | 40 |
|  |  | TCCTCCTAAACCATATCCTGAGT |  |  |  |
| *GRF2* | At5g37025 | CATCTTTAGCTATCTTCCTCCCA | 55 | 499 | 40 |
|  |  | GTAAGAAGGTGGAGGAATCTGG |  |  |  |
| *GRF3* | At5g37026 | AGATGAAGCAAGAAAGCAACA | 55 | 491 | 40 |
|  |  | AGGAAATTTGGATAAAAACCAAA |  |  |  |
